# Solar energy converters based on multi-junction photoemission solar cells

**DOI:** 10.1038/s41598-017-16455-6

**Published:** 2017-11-23

**Authors:** O. E. Tereshchenko, V. A. Golyashov, A. A. Rodionov, I. B. Chistokhin, N. V. Kislykh, A. V. Mironov, V. V. Aksenov

**Affiliations:** 1 Rzhanov Institute of Semiconductor Physics SB RAS, 630090 Novosibirsk, Russian Federation; 2 Novosibirsk State University, 630090 Novosibirsk, Russian Federation; 3 CJSC “EKRAN FEP”, 630060 Novosibirsk, Russian Federation

## Abstract

Multi-junction solar cells with multiple p–n junctions made of different semiconductor materials have multiple bandgaps that allow reducing the relaxation energy loss and substantially increase the power-conversion efficiency. The choice of materials for each sub-cell is very limited due to the difficulties in extracting the current between the layers caused by the requirements for lattice- and current-matching. We propose a new vacuum multi-junction solar cell with multiple p-n junctions separated by vacuum gaps that allow using different semiconductor materials as cathode and anode, both activated to the state of effective negative electron affinity (NEA). In this work, the compact proximity focused vacuum tube with the GaAs(Cs,O) photocathode and AlGaAs/GaAs-(Cs,O) anode with GaAs quantum wells (QWs) is used as a prototype of a vacuum single-junction solar cell. The photodiode with the p-AlGaAs/GaAs anode showed the spectral power-conversion efficiency of about 1% at *V*_*bias*_ = 0 in transmission and reflection modes, while, at *V*_*bias*_ = 0.5 V, the efficiency increased up to 10%. In terms of energy conservation, we found the condition at which the energy cathode-to-anode transition was close to 1. Considering only the energy conservation part, the NEA-cell power-conversion efficiency can rich a quantum yield value which is measured up to more than 50%.

## Introduction

**Figure 1:**
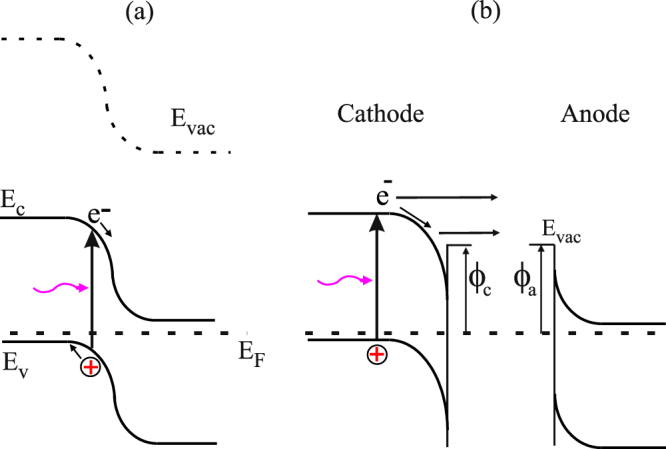
Transformation of the single p-n junction (**a**) into two electrodes (cathode and anode) activated by (Cs,O) and separated by the vacuum gap (**b**). Electrons emitted from the cathode to anode through the vacuum gap. The anode material can be chosen independently from the cathode material.

Multi-junction solar cells based on III–V heterostructures with multiple p-n junctions are known as the most efficient solar energy convertors. Using multiple bandgaps in a single heterostructure allows reducing the relaxation energy loss and substantially increases the power-conversion efficiency up to 50% in the systems with concentrators^1,2^, and up to 40% for unfocused sunlight^2–4^. However, multi-junction cells are much more difficult to produce with respect to (w.r.t.) a single-junction cell because lattice and electrical characteristics of each layer have to be carefully matched. This limits the multi-junction solar cells construction to certain materials best met by the III–V semiconductors^5–8^. One of the solutions is to use mechanically separated p–n junctions and then wire them together separately outside the cell. The transformation of a single p-n junction into two electrodes (cathode and anode) is demonstrated in Fig. 1. To transfer electrons from a cathode to an anode, it is necessary to reduce the work function of both electrodes. The work-function lowering for semiconductors is traditionally achieved by coating surfaces with cesium and oxygen (Cs,O)^9^. The cathode and anode separation by a vacuum gap forms the plate capacitor (Fig. 1b). The p-type cathode with the adsorbed (Cs,O) layer produces the so-called effective negative electron affinity state (vacuum level is below the conduction band maximum in the bulk) which allows reaching the quantum yield more than 70% for such photocathodes like GaAs, GaAsP and GaN^10,11^. One can see that the anode material selection is not dependent on the cathode and can be chosen with the requirements of the lowest work function and low resistivity. The power output of the NEA converter is calculated from the difference between cathode current *J*_*C*_, and the reverse current (photocurrent, thermionic and electron recycling) from the anode *J*_*A*_ multiplied by the operating voltage. The operating voltage is given by the contact potential difference (CPD) between the cathode and anode work functions and any extra voltage *V*_*bias*_ across the vacuum gap:

1 $${P}_{NEA}=JV=({J}_{C}-{J}_{A})({{\rm{\Phi }}}_{C}-{{\rm{\Phi }}}_{A}+{V}_{bias}),$$

if we assume *J*_*c*_ ≫ *J*_*a*_ (no anode photocurrent, almost zero thermionic and low electron recycling current), the photodiode spectral conversion efficiency at *V*_*bias*_ = 0 can be expressed:

2 $$\eta =\frac{e({\varphi }_{c}-{\varphi }_{a})}{\hslash \omega }\frac{{N}_{e}}{{N}_{ph}}=\frac{e{\rm{\Delta }}\varphi }{\hslash \omega }QY,$$

where Δ*ϕ* = (*ϕ*_*c*_ − *ϕ*_*a*_) is the CPD, e − electron charge, *N*_*e*_ and *N*_*ph*_ − number of emitted electrons and incident photons per second, respectively, and ratio *N*_*e*_/*N*_*ph*_ = *QY* is a quantum yield. From equation (2) it follows that the maximum of conversion efficiency can reach quantum yield *η* → *QY* when $e({\varphi }_{c}-{\varphi }_{a})\to \hslash \omega$. One can see in Fig. 1b that (*ϕ*_*c*_ − *ϕ*_*a*_), assuming a similar decrease of the affinity on p- and n- type semiconductors and the opposite band bending near the surfaces which is typical of wide-gap III-V semiconductors. Nevertheless, the photocurrent flows in such system. The point is that the maximal energy of emitted electrons w.r.t. the vacuum level of the anode corresponds to the difference between the conduction band minimum in the bulk of a cathode and the work function (WF) of an anode (*eϕ*_*a*_) and equals the effective NEA *χ* ^*^ in the case of *ϕ*_*c*_ = *ϕ*_*a*_. Thereat, the CPD in equation (2) should be corrected:

3 $${\rm{\Delta }}{\varphi }^{\ast }=e(({\varphi }_{c}+{\chi }^{\ast })-{\varphi }_{a})\cong {E}_{g}-e{\varphi }_{a},$$

The separation of p-n junction by a vacuum gap allows us to use the materials independent from the lattice matches, while a combination of multi-junction photoemission solar cells with various energy gaps excludes the problem of current matching. From equation (3) it is seen that CPD can be increased either by increasing the cathode WF or by decreasing the anode WF. An idea of the cathode WF increase was recently introduced in a new approach called the photon-enhanced thermionic emission (PETE), which has pointed out that the electron emission from a material, where electrons must first overcome a positive electron affinity energy barrier, could be useful for energy conversion^12,13^. PETE has been proposed for the high-efficiency sunlight to electricity conversion which is based on the electron emission from a hot semiconductor surface, assisted by the direct photonic excitation in the semiconductor. At the film surface, carriers encounter a positive electron affinity barrier, and only electrons with sufficient thermal energy can overcome this barrier and escape into vacuum. Any increase in this electron affinity raises the output voltage of the PETE device, but the increased electron affinity also results in the exponential reduction of the emission current reducing the device power conversion efficiency. In Table 1 we compare the characteristic of NEA and PETE cells which, one can see, differ by a factor of *e* ^−*χ*/*kT*^. The other problem is that the working temperature regimes of the most effective III-V photocathodes are below 100 °C, which is well below the effective PETE regime^14^. For this reason, using high efficiency NEA cathodes and low WF anodes seems more perspective in a power conversion cell. The lowest work function was reported for the phosphor-doped diamond to have the work function of 0.9 eV^15^ and, recently, it was reported even ~0.6 eV^16^, and that gives the CPD equal to Δ*ϕ* = 0.8 eV and 1.4 eV GaAs and GaAsP (Eg ≈ 2 eV) cathodes, respectively. Although PETE converters were proposed for the high efficiency sunlight to electricity conversion^12,13^, up to now only calculations have demonstrated this possibility^17^. PETE and thermionic energy converters today still fall short on the energy conversion efficiency due to physical limiting factors including: (i) radiation heat transfer between electrodes, (ii) thermal energy losses to the environment, and (iii) space charge effects which limit the electron flow across the gap. Factors (i) and (ii) are not the problem in the case of NEA converters for the reason that cathode and anode temperatures are the same and close to the environmental temperature. The negative space charge (NSC) is a well-known effect in the PETE and thermionic emission in general. The electrons emitted from the cathode form a negatively charged electron cloud in the inter-electrode space and may produce an added energy barrier that impedes emitted electrons from reaching the anode. Reducing the gap between the electrodes to a few microns was proposed to reduce the performance loss due to NSC in thermionic and PETE converters. The maximal potential barrier can be obviously reduced in a microscale vacuum gap, and larger short circuit currents can be obtained by decreasing the vacuum gap. For PETE, however, the gap should not be below several *μm* caused by the nearfield radiation heat transfer between the two electrodes and, as a result, the reduction of converter efficiency^18^. Such small gaps can be produced by the insertion of very small spacers^19,20^. The cathode and anode temperatures are practically the same in NEA convertors and the reduction of the vacuum gap between the electrodes to a few microns became possible.

**Table 1:** Comparison of NEA and PETE cathodes emission characteristics.

PETE	NEA
*j*_*e*_ = *enP*_*e*_(*ε*)〈*ν*_z_〉*e* ^−*χ*/*kT*^	*j*_*e*_ = *enP*_*e*_(*ε*)〈*ν*_*z*_〉
$QY=\frac{P{e}^{-\chi /kT}}{1+{({\alpha }_{\hslash \omega }{L}_{D})}^{-1}}$	$QY=\frac{P}{1+{({\alpha }_{\hslash \omega }{L}_{D})}^{-1}}$
$$\begin{array}{c}\eta =\frac{e({\varphi }_{c}-{\varphi }_{a})}{\hslash \omega }\frac{{N}_{e}}{{N}_{ph}}{e}^{-\chi /kT}\\ =\frac{e{\rm{\Delta }}\varphi }{\hslash \omega }{e}^{-\chi /kT}QY\end{array}$$	$$\begin{array}{c}\eta =\frac{e({\varphi }_{c}-{\varphi }_{a})}{\hslash \omega }\frac{{N}_{e}}{{N}_{ph}}\\ =\frac{e{\rm{\Delta }}\varphi }{\hslash \omega }QY\end{array}$$

In a PETE cathode, the total emitted current density is *j*_*e*_ = *enP*_*e*_(*ε*)〈*ν*_*z*_〉*e* ^−*χ*/*kT*^ (*), where e – is the electron charge, n − is the total electron concentration in the conduction band, factor *P*_*e*_ represents the probability that an electron reaching the surface has sufficient thermal energy $\sim kT$ to overcome the electron affinity barrier *χ* = *E*_*vac*_ − *E*_*c*_ and escape into vacuum, 〈*ν*_*z*_〉 is the average magnitude of an electron thermal velocity perpendicular to the surface. ${\alpha }_{\hslash \omega }$ is the absorption coefficient of the photocathode material, and *L*_*D*_ is the electron diffusion length. From equation (2) it can be seen that the QE is influenced by *P* and *L*_*D*_ simultaneously, and both of the two parameters are limited by the p-type doping concentration.

In this work we realized a prototype of a vacuum single-junction solar cell (vacuum-diode) with semiconductor electrodes as the emitter and collector both activated to the NEA states. The photocathode can work in the transmission and reflection modes simultaneously and it demonstrates 27% and 23% of QY in the transmission and reflection modes at *V*_*bias*_ = 0, respectively, and 37% of QY under the accelerating bias of 0.5 V. The energy distributions of emitted electrons in transmission and reflection modes were measured and that allowed establishing the electron energy loss mechanisms of emitted electrons. We explore a promising strategy using a wide gap AlGaAs anode with a thin top GaAs layer (5 nm) and GaAs quantum wells (QWs) to demonstrate the potential of NEA systems for a high solar energy conversion.

## Results

**Figure 2:**
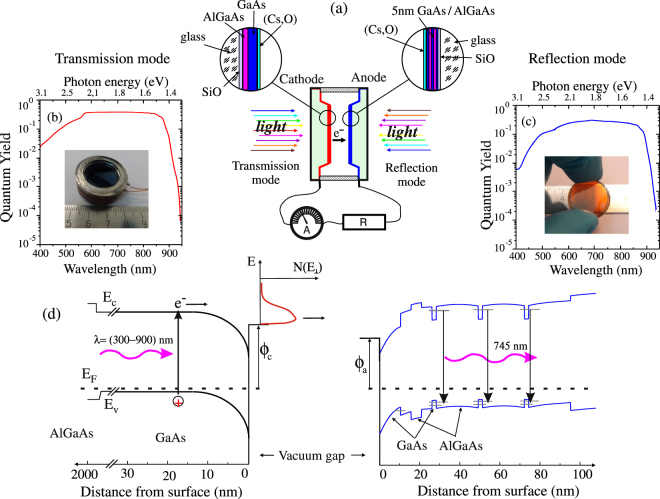
(**a**) Schematic presentation of the compact vacuum photodiode for the investigation of solar cell properties and its photo (inset in (**b**)). The insets in (**a**) show the zoomed images of the cathode and anode structures bonded to glass, which consisted of the antireflection SiO layer, AlGaAs layers, p-GaAs active layer and Cs-O activation layer. (**b**) Quantum efficiency of the photocathode as a function of wavelength under the cathode (transmission mode) illumination. The compact vacuum tube from the cathode side is shown in the inset. (**c**) The photocathode quantum efficiency as a function of wavelength under illumination through the anode (reflection mode). The anode GaAs/AlGaAs QWs structure (200 nm) bonded to glass is shown in the inset. In both (**b**) and (**c**) accelerating voltage *V* = +0.5 V was applied at the anode. (**d**) The band diagram of two semiconductor electrodes with the NEA separated by a vacuum gap and electrically connected.

Figure 2 presents some essential background information related to our approach. A planar vacuum photodiode with the zoomed images of the photocathode and anode structures bonded to glass are shown in Fig. 2a. The photocathode is a standard AlGaAs/GaAs structure with a 2.5 *μm* GaAs active layer activated by Cs and oxygen, which works as a light absorber and electron emitter. The anode is the AlGaAs/GaAs/AlGaAs quantum wells structure with a 5 nm GaAs top layer also activated by Cs and oxygen to the NEA state. The thickness of the anode structure is 200 nm. The wide gap and thinness of the anode allow working with a photodiode in both transmission and reflection modes. The quantum yield (QY) of the GaAs photocathode in transmission (t-mode) and reflection (r-mode) modes are shown in Fig. 2(b) and (c), respectively. In the insets are the image of the compact vacuum tube from the cathode side (Fig. 2b) and the anode heterostructure bonded to glass (Fig. 2c). The maximum of the QY obtained by activating the cathode with (Cs,O) amounted 37% at 700 nm. The maximum of QY can be spectrally shifted by the emission layer thickness optimization. This optimum depends on the absorption length and the electron diffusion length, which means that most of the photons with different wavelengths should be absorbed in the emission layer and the photogenerated electrons can reach the surface within their diffusion length. Therefore, the thickness *d* of the GaAs emission layer should follow: *α* ^−1^< *d* < *L*, where *α* ^−1^ is the absorption coefficient and *L* is the diffusion length. For our cathodes, minority carrier diffusion length *L* is about 5 *μm* for the p-type GaAs with hole concentration 5×10^18^ *cm* ^−3^.

**Figure 3:**
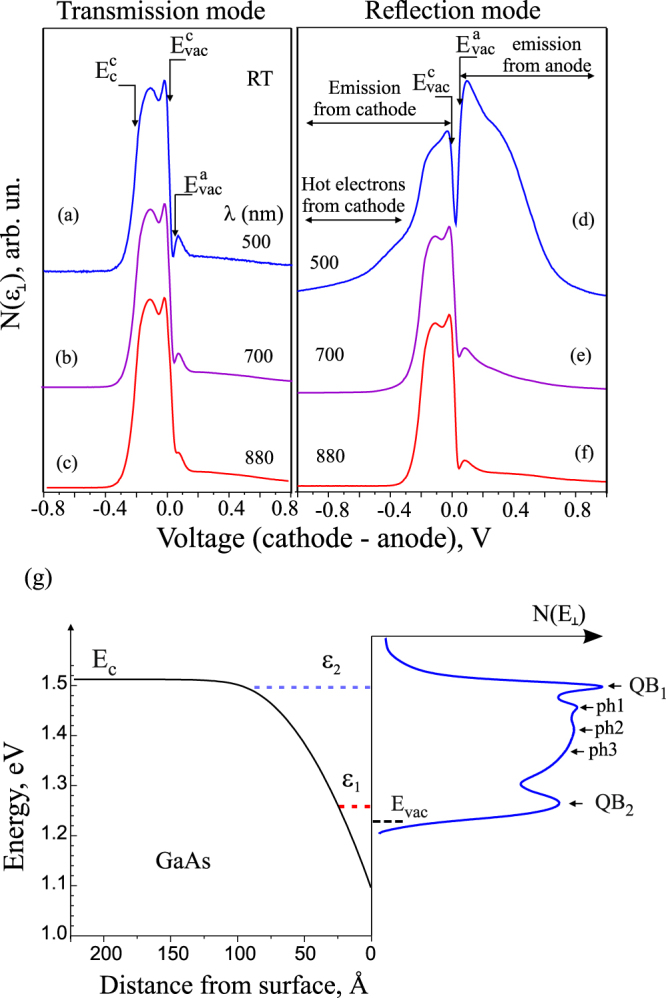
QY spectra for photocathode (t-mode) and photoanode (t-mode) under applied accelerating 1 V for the respective electrode. Energy distribution of emitted electrons N(E_⊥_) in transmission (**a**–**c**) and reflection (**d**–**f**) modes w.r.t. the GaAs cathode for 500, 700 and 880 nm excitation wavelengths at 300 K. The energies corresponding to the vacuum level *E*_*vac*_ and to the bottom of the conduction band *E*_*c*_ are indicated by the vertical arrows. The negative retarding voltage corresponds to the EDC from the NEA GaAs cathode, while the positive from the NEA QW anode. (**e**) The downward band bending of the conduction band at the surface of p-GaAs (Cs,O), determined from the calculated subband positions and measured EDC from NEA GaAs cathode, is shown on the right side at 20 K. The two peaks observed in the measured EDC correspond to the electron emission from quantized conduction-band subbands with energy *ε*_1_ and *ε*_2_.

The band diagram of the two semiconductor electrodes separated by a vacuum gap and electrically connected is shown in Fig. 2d. In an ideal case of emission, one can expect the observation of only a narrow emission peak from the bulk conduction band with the width of the order of $\sim kT$. In the point of the fact, due to the electron scattering in the emission process, the energy distribution width is about ~*χ* ^*^. The energy distribution of emitted electrons provides important information on the electron energy loss mechanism during emission and it demonstrates a powerful example of the NEA potential for power conversion. The energy distribution curves (EDC) of emitted electrons *N*_⊥_(*ε*) in transmission and reflection modes for the three excitation wavelengths measured at 300 K are shown in Fig. 3(a–f). Note that the horizontal axis represents the voltage applied to the cathode-anode (directly related to the electron energy), where it is seen that, at zero potential, the photocurrent flows in a wide spectral range. It is also seen that both, the cathode and anode contribute to the EDC. The EDC measured in the t-mode illumination geometry shows a very weak dependence on the photon wavelength that can be explained by a relative thick GaAs layer (2.5 *μm*) with respect to the absorption length (≤1 *μm*). For electrons that are reflected from the anode, with some energy loss being captured, return to the cathode with the energy above the cathode vacuum level. These electrons first occupy the conduction band from which they can be re-emitted again. This form of electron recycling makes NEA converters, as it was shown^21^, less susceptible to a negative space charge loss.

For the r-mode illumination geometry, the EDC in the 900–760 nm range is similar to those measured in the t-mode because of the anode transparency in this wavelength range. A decrease in the wavelength is accompanied by an increase in the width of the high-energy tail of the cathode-EDC and an increase of the anode-EDC amplitude. For the cathode-EDC, this observation is expected because, at higher photon energies, the majority of photoelectrons with excess kinetic energy are generated in a close proximity to the emitting surface and, as a result, a significant number of non-thermalized electrons are emitted before thermalization, and their contribution to the photocurrent increases in tandem with the increase in the photon energy. The amplitude of anode-EDC increases for a wavelength shorter than 740 nm, caused by the photon adsorption in QWs and the AlGaAs matrix. The anode-EDC width is 0.5 eV and wider than those of the GaAs-cathode (0.3 eV), and that can be explained by a lager band bending value and the effective NEA prepared on the wider gap anode electrode.

The temperature reduction of the emitter is known to reduce the temperature broadening and allow elucidating the microscopic scattering mechanisms. The electron energy distribution measured for the p^+^−GaAs(Cs,O) transmission-mode photocathode at 20 K is presented in Fig. 3(g). The most interesting EDC features observed at low temperature (LT) are marked as *QB*_1_, *QB*_2_ and *ph*_1_ − *ph*_3_, shown in Fig. 3(g). Peaks *QB*_2_ and *QB*_1_ are located 20 ± 1 meV and 228 ± 3 meV below the conduction band bottom in the semiconductor bulk and correspond to the electron emission from the quantized conduction-band subbands with energy *ε*_1_ and *ε*_2_. The band diagram with the downward band bending at the surface of p−GaAs(Cs,O), determined from the calculated subband positions and measured EDC from NEA GaAs cathode, is shown on the left side of Fig. 3(g). The shape of the *QB*_2_ peak in the vicinity of its maximum is described well by the Gaussian profile with a half-width of 30 meV. Features *ph*_1_ − *ph*_3_ are observed on the low-energy side of peak *QB*_2_. The energy gaps *QB*_2_ − *ph*_1_, *ph*_1_ − *ph*_2_ and *ph*_2_ − *ph*_3_ between the features turned out to be approximately the same and equal to 42 ± 1 meV. This value exceeds the energy of the long wavelength longitudinal optical phonons $\hslash \omega (LO)\,=\,36.7$ meV in GaAs. Peak *QB*_2_ corresponds to the elastic emission, and peaks *ph*_1_ − *ph*_3_ correspond to the inelastic electrons emission from the upper *QB*_2_ subband of the two-dimensional quantization band in the near-surface potential well into vacuum with the emission of one, two and three phonons, respectively. The peak at *ph*_3_ is hardly visible in EDC, but it appears in the derivative. A more detailed analysis of the emission mechanism from the semiconductor photocathodes with NEA will be published elsewhere.

**Figure 4:**
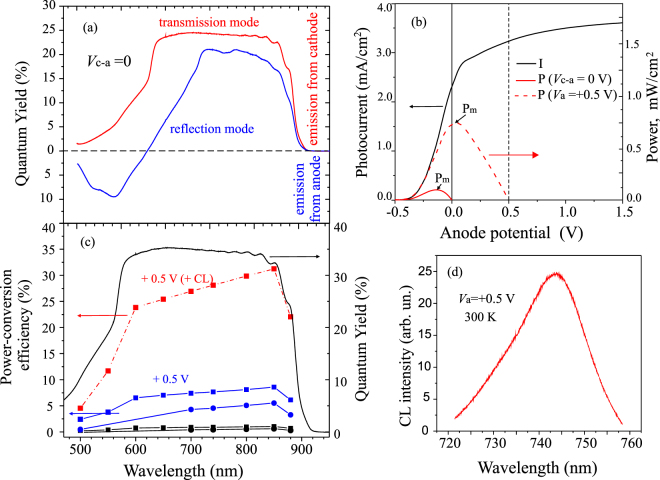
Photodiode quantum efficiency of in the transmission and reflection modes at *V*_*b*_ = 0. (**b**) *I*–*V* and *P*–*V* curves for the 800 nm excitation. (**c**) Power-conversion efficiency measured for *V* = 0 (black squares and circles for transmission for transmission modes, respectively) and for *V* = +0.5 V (blue squares and circles for transmission and reflection modes, respectively). Red squares (dashed line) indicate the measured power-conversion efficiency for the *V* = +0.5 V anode bias considering the energy conservation in the cathodoluminescence emission process from the anode (**d**).

According to equation (3) the power-conversion efficiency is proportional to QY and that gives an idea to study first the spectral quantum efficiency in both transmission and reflection modes at the zero applied voltage between semiconductor electrodes. The QYs for t- and r- modes are shown in Fig. 4(a), and one can see that, at *V* = 0, the QY reduces w.r.t. the accelerating potential. This reduction is caused by the space charge effect and, probably, by a stronger scattering of low-energy electrons with energies close to *E*_*vac*_. Nevertheless, the cathode QY reaches 27% in the t-mode and more than 20% in the r-mode. The solar cell behavior is represented by the current versus voltage curve. Typical *I*−*V* (solid) and *P*−*V* (dashed) curves of the photodiode in the t-mode, from which the maximal power-conversion efficiency can be calculated, are shown in Fig. 4(b).

Open-circuit voltage *V*_*oc*_ is the maximal voltage available from a solar cell, and this occurs at the zero current. The open-circuit voltage corresponds to the amount of the forward bias on the solar cell due to the bias of the solar cell junction with the light-generated current and equals 0.3 V in the t-mode and it can reach 0.6 V in the r-mode, as can be seen from the retarding potential for the hot electrons demonstrated in Fig. 3(d). *V*_*oc*_ depends on the saturation current of the solar cell and the light-generated current and it is then a measure of the amount of recombination in the device. The maximal power point is the point on the *I*−*V* solar cell curve corresponding to the maximal output electrical powers *P*_*m*_ = *I*_*m*_ *V*_*m*_.

The power-conversion efficiency equation of both NEA GaAs photocathode modes can be calculated from the ratio of the power take-off maximum to the light absorption power as follows:

4 $$\eta =\frac{{P}_{max}}{\hslash \omega {N}_{ph}}=\frac{{U}^{\ast }{I}^{\ast }}{\hslash \omega {N}_{ph}}=\frac{{e}^{2}{U}^{\ast }}{\hslash \omega {I}^{sat}}QY,$$

where we used relation *N*_*ph*_ = *N*_*e*_/*QY*, *I* ^*sat*^ - saturation current taken from *I*−*V*, *I* ^*sat*^ = *eN*_*e*_. The measured spectral power efficiency is about 1% for the t-mode and 0.7% for the r-mode in the 550–880 nm spectral range, as shown in Fig. 4c. Note that this efficiency is obtained for the cathode-anode system in which both electrodes are p-type semiconductors. As a result, the fill factor is rather low ≈0.3. If we assume an anode with a reduced work function, for example, by 0.5 eV (dashed vertical line at 0.5 V), the power conversion increases by almost one order of magnitude, shown by the red dashed curve in Fig. 4(b) and by the blue curves in Fig. 4(c).

**Figure 5:**
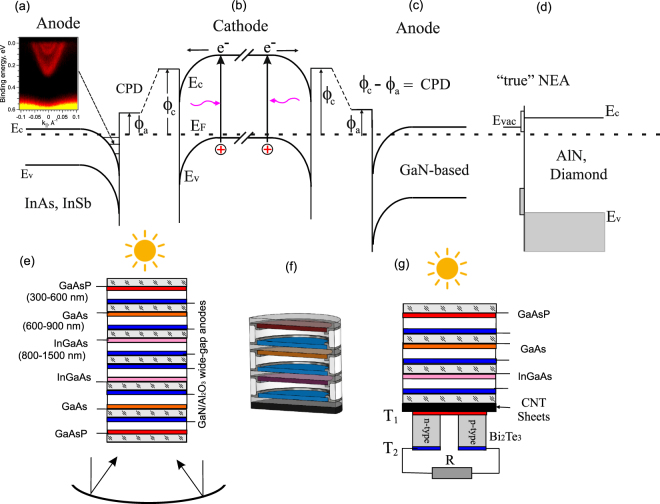
Band diagram of semiconductor electrodes with the NEA separated by a vacuum gap for the light absorber cathode (**b**) and narrow gap semiconductor (InAs, InSb) anode (**a**) and wide gap semiconductor (GaN) anode (**c**) or “true” NEA (diamond, AlN) anode (**d**). Possible constructive schemes of multi-junction photoemission solar cell (**f**) absorbing the light from both sides of the NEA cell (**e**), and NEA converter combined with an infrared solar thermoelectric converter (**g**).

Maximizing the total power is the goal of the solar cell design. Multi-junction photovoltaics, as compared to single-junction cells, is known to have reduced currents, but, at the same time, the excited electrons are more energetic and have a greater electric potential. So, the current reduction is compensated by an increase in voltages, and the overall cell power is higher. A relative low efficiency of the presented photodiode is limited by a small difference in Δ*ϕ* ^*^ (equation (3) which is determined using the p-type anode. One can see from the band diagram, shown in Fig. 1(d), that the electrons injected into the anode lose their energy reaching the Fermi level either from the vacuum level or conduction band in the anode bulk. The possible way to reduce energy losses will be discussed in Fig. 5. For this vacuum photodiode, we can try to estimate the maximal possible efficiency by measuring the cathodoluminescence from the anode and assuming that we can use the emitted light power in the positive work. The power conversion of CL is, in principle, equivalent to the upward Fermi level shift from the p − to n − type position. If we apply the 0.5 V anode bias, the cathodoluminescence from the anode is detected at the maximum of 745 nm (1.66 eV), as it is shown in Fig. 4(d). Thus, we spend the energy of 0.5 eV for electron acceleration, but gain 1.66 eV in the CL conversion. Assuming the electron − photon conversion equal to 1, equation (2) can be modified as

5 $$\eta =\frac{e{\rm{\Delta }}{\varphi }_{c}+1.66\,eV}{\hslash \omega +0.5\,eV}QY,$$

and it is shown in Fig. 4(c) (filled red squares). One can see that, for the absorbed photons close to the cathode energy gap, the power conversion efficiency is close to QY. Note that, in the modern photocathode technology, QY exceeds the value of 50%. This result shows that the CPD value plays a crucial role in the solar cell conversion efficiency. Regarding this problem, the interesting and important question in this context is how to increase the difference in CPD: Δ*ϕ* = (*ϕ*_*a*_ *−* *ϕ*_*c*_)? For GaAs electrodes the theoretical CPD is equal to the energy gap value (1.4 eV). In practice, the n-type GaAs surfaces in vacuum, as well as most of n-type semiconductors, exhibit the upward band bending (0.7–0.9 eV for n-GaAs) at the surface that diminishes the effect from various Fermi level positions in the electrode bulks. On the other hand, it gives an idea for searching the semiconductor materials with the surface properties allowing changing both the electron affinity and surface potential (band bending).

Among the promising anode candidates are the narrow gap III-V semiconductors (InAs, InSb). The result of experimental Angle-Resolved Photoemission Spectroscopy (ARPES) result on the clean InAs(111)A-(1 × 1) surface, which demonstrates the presence of the 2D quantized electron gas near the surface caused by downward band bending at the surface for the n-type doped semiconductor, is shown in Fig. 5(a). Taking into account the electron affinity 4.5–4.9 eV on the InAs surface^22^, which depends on surface termination and reconstructions, and ~3.5 eV work function reduction under Cs and oxygen adsorption, one can evaluate a minimal absolute value of the WF on the (Cs,O)/InAs surface equal to 0.7 eV that gives the CPD Δ*ϕ* ^*^ ≅ 0.7 eV in case of the GaAs cathode and even a higher CPD value for wider gap semiconductor cathodes.

For the anode, it is also possible to use the wide gap GaN-based semiconductors^23^ (Fig. 5(c) or, so called, “true” NEA materials (Fig. 5(d) which also allow working in both transmission and reflection geometries. The work function and the electron affinity of clean GaN(0001) surfaces were determined as 3.9 eV and 3.35 eV, respectively^24^. Under the Cs adsorption, the decrease of WF down to 1 eV was found and shown to be due to the formation of a charge accumulation layer in the near-surface region.

Multiple materials with bandgaps that expand the solar spectrum are used in the highest-efficiency solar cells. Finally, we propose new vacuum multi-junction solar cells with multiple p-n junctions separated by a vacuum gap that allows using semiconductor materials with various gaps schematically shown in Fig. 5(e,f). Photoemission multi-junction solar cells consist of separated single-junction solar cells following each other so that each cell going from the top to the bottom has a smaller bandgap than the previous one, and so that it absorbs and converts the photons that have energies greater than the bandgap of that layer and less than the bandgap of the previous cell and wires them together separately outside the cell. The solar element can be constructed in a way to absorb the light from both sides of the NEA cell, as it is shown in Fig. 5(e), by using reflectors. This NEA cell is effective in the wavelength range of 300–1300 nm. The infrared absorption edge in NEA emitters is about 1300 nm and limited by the energy gap of InGaAs on which the NEA state is possible. A further enhancement in efficiency can be obtained by combining a NEA converter with a solar thermoelectric conversion based on Bi_2_Te_3_ and Carbon Nanotube Composites^25^ shown in Fig. 5(g). Bi_2_Te_3_, a typical thermoelectric material with high thermoelectric conversion efficiencies, recently demonstrated a possibility of creating a built-in p−n junction in a single crystal during the growth with high Seebeck coefficient values^26^. It was proved that carbon nanotubes possess a very high light absorption coefficient in a very broad spectrum, showing a very good potential for solar-thermal conversion.

We should point out that the results reported here are the primary contributions on NEA solar cells. We believe that there is still a large potential for a further optimization in QY of NEA emitters and the increase of energy conversion efficiency following the tendency *η* → *QY*. The most challenging aspect is finding the anode material, where the requirements of a very low work function and low resistance need better solutions. From the results shown here, the concept of harvesting photon in a multi-junction solar cell and a thermal energy convertor (through infrared thermoelectric conversion) together is highly attractive for improving the efficiency of the solar-energy collection that deserves a serious investigation especially for space power systems.

## Methods

The experiments were carried out in a planar vacuum photodiode consisting of the GaAs(Cs,O) photocathode on the glass substrate from one side and the AlGaAs/GaAs/AlGaAs(Cs,O) structure with the QW anode on the glass substrate from the other one, which are hermetically fixed parallel to each other at the opposite ends of an aluminum-oxide ceramic cylinder^27^. The diameters of the cathode and anode were 20 mm, with the gap between the electrodes of about 1 mm. The final step of the cleaning procedure is made inside a glove box, flooded with pure nitrogen, where the cathodes are chemically treated in a solution of HCl in isopropanol^28^. Apart from removing oxides, this leaves the GaAs surface As-rich, i.e., with a top As layer of about 1–2 monolayer thick, which is later removed by heating the cathode and anode in vacuum^29^. The transfer energy distribution curves for the emitted photoelectrons were measured by applying a potential between the photocathode and anode. The photoelectron spectra were measured by differentiating delay curves using the lock-in technique. The collector current is detected using the lock-in technique, slowly ramping the retarding voltage *V*_*R*_ over the relevant electron energies and additionally modulating it with the amplitude of 1 mV at the typical frequency of 140 Hz. The resulting lock-in output signal is proportional to the electron beam energy distribution curve.
